# The Value of a Comparative Approach to Understand the Complex Interplay between Microbiota and Host Immunity

**DOI:** 10.3389/fimmu.2017.01114

**Published:** 2017-09-14

**Authors:** Norma M. Morella, Britt Koskella

**Affiliations:** ^1^Department of Integrative Biology, University of California, Berkeley, Berkeley, CA, United States

**Keywords:** timing of exposure, microbiome, defensive symbiont, microbiome transmission, microbiome variation

## Abstract

The eukaryote immune system evolved and continues to evolve within a microbial world, and as such is critically shaped by—and in some cases even reliant upon—the presence of host-associated microbial species. There are clear examples of adaptations that allow the host to simultaneously tolerate and/or promote growth of symbiotic microbiota while protecting itself against pathogens, but the relationship between immunity and the microbiome reaches far beyond simple recognition and includes complex cross talk between host and microbe as well as direct microbiome-mediated protection against pathogens. Here, we present a broad but brief overview of how the microbiome is controlled by and interacts with diverse immune systems, with the goal of identifying questions that can be better addressed by taking a comparative approach across plants and animals and different types of immunity. As two key examples of such an approach, we focus on data examining the importance of early exposure on microbiome tolerance and immune system development and function, and the importance of transmission among hosts in shaping the potential coevolution between, and long-term stability of, host–microbiome associations. Then, by comparing existing evidence across short-lived plants, mouse model systems and humans, and insects, we highlight areas of microbiome research that are strong in some systems and absent in others with the hope of guiding future research that will allow for broad-scale comparisons moving forward. We argue that such an approach will not only help with identification of generalities in host–microbiome–immune interactions but also improve our understanding of the role of the microbiome in host health.

## Introduction

Across kingdoms of life and branches of immunity, there are conserved characteristics in how hosts interact with their microbiome. Plants, mammals, and invertebrates are all able to differentiate between self and non-self, where they tolerate, and in some cases promote, associations with commensal or beneficial microbes while retaining the ability to sense and attack microbial pathogens. In many cases, beneficial microbes can even be considered an extension of the immune system through either competitive exclusion of pathogens or direct inhibition of their growth. Furthermore, non-pathogenic microbiota can both interact with and influence the adaptive and innate immune systems. Across these diverse host systems, the evidence for an interaction between the microbiome and immunity is strong and unsurprising given that eukaryotic evolution has occurred entirely within a microbial world. The topic of immunity is highly complex and may seem inaccessible to those outside the discipline. However, from the perspective of evolutionary ecology, there is much that can be learned about host–microbe adaptation and coevolution through exploring topics in immunity. Therefore, our goal in this perspective piece is to broadly examine the key characteristics of known interplay between host immune systems and symbiotic bacteria across well-studied systems (the more detailed aspects of which, including microbiome variability among individuals, stability over time, mode of transmission, and evidence for host–microbiota co-speciation, we summarize in Table 1). We focus on the bacterial component of the microbiome but recognize the importance of fungal members and viruses, especially bacteriophages, given their known impact on the microbiome [e.g., Ref. (1, 2)] and possible role in host immunity [e.g., Ref. (3)].

**Table 1:** Characteristics of host/microbiota association.

Host	Site/organ	Core taxa	Max O.T.U. range^a^	Variability between individuals		
				Early development	Adult	
Humans	Skin	4 phyla	14–182	May depend on delivery method in first weeks	Yes: more similarity between sites on one body than between bodies; variation between bodies depends on skin site	
	Gut	3 phyla	237–395	Lower alpha diversity than adults; higher variability between individuals	Yes: tend to fall into three enterotypes	
	Oral	6 phyla	600–19,000	Lower alpha diversity than adults; may depend on delivery method in first weeks	Low at genus level; higher at species level; varying results across studies	

Honey bee (*Apis mellifera*)	Gut	3 phyla; 6–10 species	68–99	Bacteria limited or absent in larvae/newly emerged bees	No: core species across geography, “tasks,” diets, and time	
Termite	Gut	11 phyla	357–5413	Lower alpha diversity than adults, limited larval studies	Dominated by some phyla, but very diet dependent	
Aphid	Gut	1 primary species	3–67	?	Primary symbionts: low; secondary: varies with geography or host plant	
*Drosophila melanogaster*	Gut	2–4 genera	21–122	High	Primarily same genera but varies based on diet and wild/lab strain	
Tsetse fly (*Glossina* spp.)	Various (Gut, bacteriome, milk gland organ)	3 phyla; 3 primary species	25 (one study)	Some primary symbionts: low; secondary: ?	Some primary symbionts: low; secondary: variable, but limited studies	

*Arabidopsis thaliana*	Rhizosphere	3–7 phyla	778–1,262	?	Soil and/or genotype driven	
*Arabidopsis thaliana*	Rhizosphere	4 phyla	840–5,057	?	Abundance variable; may converge over time	
Maize	Phyllosphere	4 phyla	396–61,067	Abundance/diversity variable between genotypes; high consistency with synthetic community	Genotype, soil type, geography driven	

**Host**	**Site/organ**	**Heritability of microbiota**	**Evidence for role of host genetics shaping microbiota**	**Transmission**	**Temporal stability**	**Co-speciation**

Humans	Skin	Yes	Yes	Maternal, contact, environmental	Skin site dependent; stability shown up to 2 years	Yes
	Gut	Yes	Yes	Maternal, environmental	May stabilize after adolescence; diet has an impact	?
	Oral	Yes	?	Maternal, environmental	May stabilize after adolescence	?

Honey bee	Gut	Yes	?	Social hive interactions within 3 days	Change in abundance	Limited evidence
Termite	Gut	Yes	?	Early social exchange/exposure *via* proctodeal trophallaxis	Stable; diet has an impact	Yes
Aphid	Gut	Primary: yes	Primary: yes; secondary: ?	Primary: ovarian transmission; secondary: vertical or horizontal	Primary: low; secondary: may vary over time	Primary: yes
*Drosophila melanogaster*	Gut	Yes	Yes	Larval ingestion of bacteria-coated egg shells	Composition and density change with gut development and age	Maybe with endosymbionts
Tsetse Fly (*Glossina* spp.)	Various (Gut, bacteriome, milk gland organ)	Primary: yes	Limited	Primary: maternal milk, germline; secondary: ?	?	Primary: yes
*Arabidopsis thaliana*	Rhizosphere	?	Yes	Horizontal: soil	Changes with developmental stage of plant; may converge after senescence	?
*Arabidopsis thaliana*	Rhizosphere	?	Yes	Horizontal: air, soil	Communities may converge over time	?
Maize	Phyllosphere	Yes	Yes	Vertical: seed; horizontal: soil	Known successional dynamics	?

**Host**		**Site/organ**		**Reference**		

Humans		Skin		(4–10)		
		Gut		(10–19)		
		Oral		(5, 20–29)		

Honey Bee *(Apis mellifera)*		Gut		(30, 31–36)		
Termite		Gut		(37–42)		
Aphid		Gut		(43–58)		
*Drosophila melanogaster*		Gut		(59–72)		

Tsetse Fly (*Glossina* spp.)		Various (Gut, bacteriome, milk gland organ)		(73–83)		

*Arabidopsis thaliana*		Rhizosphere		(84–89)		
*Arabidopsis thaliana*		Rhizosphere		(90–94)		
Maize		Phyllosphere		(34, 95–102)		

*^a^Described in literature as 97–99% OTU cutoff or “phylotype.”*

*? Unknown or unproven*.

*“Primary” is not used to explicitly indicate “obligate” in this table*.

The microbiome field is expanding rapidly, and doing so across systems, such as plants, mouse models, humans, and insects. We suggest that taking a broad comparative approach across the diverse mechanisms of immunity and host systems could offer unique insight to how host defenses are shaped by and shape the microbiome. Such an approach can, for example, help identify areas in which research is strong for certain systems but lacking in others. Here, we emphasize areas lacking in plant host systems, but which would likely elucidate important aspects of plant health and resilience against pathogens. Filling in such gaps across systems would allow for more powerful comparative studies and may inform predictions about host–microbe adaptations in light of larger issues such as antibiotic overuse and the spread of agricultural pathogens in a changing climate.

## Overview of Host Immune Systems

To begin, we offer a brief description of immunity in mammals, plants, and insects focusing primarily on the aspects of these systems that directly relate to known interactions with the microbiome [thorough and more discipline-specific descriptions of these immune systems exist elsewhere (103–106)]. The adaptive immune system is thought to have arisen in jawed fish ≈500 million years ago (107), whereas the innate immune system likely dates back to early eukaryotic cells themselves (105, 108,). As microbial communities greatly predate the existence of multicellular eukaryotes, both branches of the immune system, therefore, evolved in the presence of microbes, and it follows that tolerance for commensal or mutualistic microbiota (those associated with hosts, but which do not cause disease) must have been a key factor in shaping the evolution of immunity. Innate immunity, found across all kingdoms of life, is largely non-specific and responds broadly to “non-self” cells. Its hallmarks include protective physical barriers and general pattern recognition receptors that sense non-self signals known as microbe-associated or pathogen-associated molecular patterns (MAMPs/PAMPs) and elicit generalized host responses (such as phagocytic ingestion of invading cells in animals or a hypersensitive response in plants). Adaptive immunity is unique to vertebrates and responds to specific pathogens through detection of antigens *via* somatically generated receptors and specialized white blood cells (B and T cells). Cellular recognition of a specific pathogen leads to clonal expansion of the lymphocyte, resulting in daughter cells that produce the same antigen-specific antibodies. Memory cells are also produced, resulting in specific and long-lasting immunological memory. Other versions of adaptive immunity may exist (discussed below), but broadly speaking, adaptive immune responses are highly specific to particular pathogens or antigens, and the immune response changes over the course of a host’s lifetime.

In many cases in vertebrates, innate immunity is the first line of defense that elicits an adaptive immune response (103), and the two systems work cooperatively to combat infection. In comparison, plants rely on an innate immunity consisting of two primary responses to microbes (106). The first branch of the immune system recognizes MAMPs/PAMPs, such as flagellin and lipopolysaccharides (LPS), through the use of transmembrane pattern recognition receptors and results in pattern-triggered immunity. However, many plant pathogens have evolved to overcome these defenses through the use of effectors. Plants with resistance genes for specific pathogens can detect the effectors through NB-LRR proteins, which represent the second response to microbes: effector-triggered immunity. In addition, plants have physical barriers to infection such as cell wall defenses (109) and can also secrete antimicrobial peptides to ward off infection (110). Insect immunology shares characteristics with both plants and mammals; responses to microbial pathogens are highly diverse among host species, but most are considered innate. Immune responses include production of antimicrobial peptides, pattern recognition receptors, and responding to pathogens *via* circulating phagocytic cells. Evidence accrued over the last few decades also shows responses reminiscent of adaptive-type immunity, such as immunological memory *via* virus-derived complementary DNAs that generate systemic immunity (111) and highly specific immune priming both within and across generations (112), but the extent of such adaptive-type immunity and similarity to vertebrate defenses remains an open question in the field (113, 114). Taking into account the type of host immunity is essential when making hypotheses about adaptation and coevolution between host and microbiota. For example, in contrast to adaptive immunity, the innate immune response is a general resistance that can only respond to selection across host generations and not within, an important distinction when considering how plants might adapt in response to microbiota as compared to vertebrates.

As is becoming increasingly evident, the immune system influences both the composition and abundance of non-pathogenic microbiota in addition to its well-studied role in preventing pathogen establishment. In mammals, this is best studied in the gut microbiome, where differentiating between these diverse symbionts and colonizing pathogens is clearly a complex problem. The human immune system maintains a homeostatic relationship with commensal microbiota through mechanisms that include stratification and compartmentalization of the intestine, production of a mucous layer and antimicrobial proteins, and limiting epithelial exposure and immune response (115), and through antibody targeting, which can limit bacterial spread and virulence, among other mechanisms (116). Interactions between the immune system and microbiota in the gut is a heavily studied field (115, 117–121), but we are still learning the ways in which aberrations in cross talk can cause or contribute to conditions, such as inflammatory bowel disease, obesity, and even certain types of cancer (122–126).

In insects, immune system responses also contribute to homeostasis with endosymbionts, reviewed in Ref. (127, 128), and restriction of other commensal bacteria to specific host compartments, as in the gut symbionts of termites (129), bees (32, 33), drosophila (130), and aphids (43), may also help maintain invertebrate symbiotic communities. The plant immune system is also critical in shaping the non-pathogenic microbiome [recently reviewed by Zipfel and Oldroyd (131)]. Two studies in *Arabidopsis thaliana* demonstrate that disrupting components of the plant immune system, such as the signaling molecules: salicylic acid (SA) and jasmonic acid (JA), influences microbial community composition: the first shows evidence for altered root microbiome communities in plant hosts lacking genes controlling production of SA compared to control plants (132) and the second shows altered microbial communities in plants with mutations in genes controlling ethylene response (another signaling molecule) and cuticle formation (90). Recent work in wheat also demonstrates a role for JA in shaping composition of the microbiome, and again in this case, activation of JA signaling pathways altered microbial diversity and composition of root endophytes (133). However, the importance of resistance genes and diversity, as well as the number of pattern recognition receptors, in shaping the plant microbiome remains an open question.

## Importance of Microbiota in Shaping Host Immunity

The interaction between the microbiome and the immune system is far from one-sided, as has been elegantly demonstrated in studies from germ-free mice. Microbiome establishment influences levels of circulating myeloid cells, macrophages in tissues, and proper functioning of innate lymphoid cells, all critical for a healthy immune response (134–136). Furthermore, microbiota is critical in development and function of components of adaptive immunity, such as B and T cell diversity and differentiation (119, 137) and there is evidence from germ-free mice supporting a role in natural killer cell priming and function (137, 138). In insects, microbes also play a role in immune system development. For example, tsetse flies lacking their vertically transmitted symbionts are immunocompromised through both altered expression of immunity-related genes and reduced levels of hemocytes, which play an important role in invertebrate immunity (83, 139–141). Altered gene expression and other physiological effects were also found in axenically raised *Drosophila melanogaster* (61). In plants, symbiotic bacteria influence host immunity by priming the plant for future exposure to pathogens through the induction of a systemic response, causing broad-range basal levels of protection. A primed plant can respond more rapidly and strongly to pathogen invasion through a variety of mechanisms, including quicker closing of stomata, less sensitivity to bacterial manipulation of defenses, upregulation of defense-related genes, and a stronger SA-related immune responses (142). In some cases, the effects of priming can even be trans-generational through chromatin and histone modification, where the subsequent generation of primed plants exhibits enhanced resistance to bacterial, fungal, and herbivorous pathogens (143–146). Immunological priming by microbiota is also observed in arthropods, where it is often described as functional adaptive immunity, as it can occur within one generation or trans-generationally. Its effects have been observed in bumble bees (147, 148), beetles (149), daphnia (150), moths (151), and many more [summarized by Contreras-Garduño et al. (152)].

Host-associated microbiota can also directly influence host resistance against invading pathogens. Common in insects and also plants and mammals, the microbiome can serve a protective role that is independent of the host immune system through antagonism, competitive exclusion, or physical exclusion of pathogens, collectively referred to as defensive symbiosis (153, 154). For example, the mammalian skin microbiota is known to play a large role in pathogen recognition and infection prevention through amplification of immune responses (155) and production of antimicrobials (156). When germ-free mice were inoculated with gut microbiota from a non-mouse host source, they showed a decreased ability to fight infection against *Salmonella*, and particular bacterial strains seem to be required for normal adaptive immune response (157). More recently, it has been shown that a mildly pathogenic bacterium of *Caenorhabditis elegans* can evolve over time to protect its host against the more virulent pathogen, *Staphylococcus aureus* (158). The importance of such pairwise interactions have been demonstrated many times [reviewed in Ref. (159, 160)], and indeed has motivated many current biocontrol strategies, but an open question in the field is how such microbe-mediated protection might scale up to the whole microbiome level. This leads to the idea that by directly protecting their host against pathogens, microbiota could hinder the evolution of host resistance by relaxing selection on host populations and, therefore, increasing host reliance on the microbiome.

## Microbiome Transmission and Timing of Exposure

In mammals, it is clear that early exposure to microbes is crucial to the development of both branches of the immune system (161), influencing not only immune development and response against pathogens but also tolerance to commensal or mutualistic microbiota (162). For example, pregnant female mice treated with antibiotics have been shown to have offspring with not only a depauperate microbiome but also decreased levels of blood neutrophils and precursor cells, resulting in higher susceptibility to infection and increased mortality rates as compared to control mice (163). In line with this, there is increasing evidence for a crucial window of opportunity for exposure to microbiota (135). A study in germ-free mice showed that introducing a healthy microbiome to adult germ-free mice did not restore normal levels of invariant natural killer T cells nor did it lessen the physical effects of induced colitis (164), and altered exposure to bacterial species and their LPS subtypes in human infant guts may have lasting and detrimental effects on development of immunity (165). In the human neonate airway, disruption of microbiome formation as early as the first 2 weeks of life can result in lifelong susceptibility to allergic airway inflammation (166). There are additional documented links between dysbiosis of early-life microbiota and disease or health conditions later in life, reviewed elsewhere (167). Despite the accruing evidence from human and mouse systems, there has been little to no exploration of such a window of opportunity for microbiome–immune system interactions in other systems, such as plants or insects. It also remains unclear whether such early exposure effects should be limited to organisms with adaptive immunity or whether priming of innate immunity at different host developmental stages also affects host–microbiome interactions.

The clear role of early exposure to microbiota, at least in mammals, suggests that it would be advantageous for a community of beneficial microbes to be transmitted vertically from parent to offspring (e.g., through direct contact at birth, seeds, or transovarian) from generation to generation. Vertical transmission in humans may be impacted by delivery mode, as there is good evidence for differences in microbiome composition and diversity between infants delivered *via* virginal birth versus those delivered *via* cesarean sections (12, 168), but it remains controversial how long-lived such effects are (4). In insects, symbionts are known to be maintained through both vertical transmission [for example, *Buchnera* in aphids; (169)] and other transmission mechanisms such as early social interactions [observed in bees; (36)], proctodeal exchange of fluids [e.g., in termites; (170)], or larval consumption of bacteria-coated egg shells [as observed in *Drosophila*; (59, 70, 171)]. Interestingly, non-social bees (in which early social transmission of symbionts would not occur) do not seem to share the core microbiome that is observed among social bees (33).

Transmission of microbiota in plants can occur vertically through the seeds, or horizontally from the soil and surrounding environment. Plants ranging from trees to grasses are known to harbor bacteria in their seeds, many of which are reported to promote plant health (172–174). Despite this, there is no evidence that plants actively select for transmission of specific microbial communities, and there are no clear examples of adaptations to ensure seed-mediated transmission. Intuitively, vertical transmission of a microbiome or symbionts would allow for maintenance of key members of the microbial community across generations, as beneficial microbes would have primary access to both spatial niches and environmental nutrients provided by seedlings. Interestingly, plants have been shown to have differential onset of resistance to pathogens throughout their life-stages, something described as age-related resistance (ARR) or developmental resistance (175–177). However, much of the work on ARR investigates exposure and resistance to specific pathogens throughout the developmental stage of the plant and does not address if there is a window of opportunity for microbial exposure in general, as observed in mammals.

## Conclusion and Opportunities for Advancement in the Field

Unsurprisingly, that the microbiome is both shaped by and shapes the host immune system is a common feature of eukaryotes. However, the mechanisms underlying such cross talk are highly variable. Although we now have a foundation of knowledge demonstrating the microbiome’s role in immune system development and function, key-questions remain unanswered across systems. One specific area for advancement is exploring the importance of both vertical transmission and timing of microbiome exposure across systems with diverse immune mechanisms. For example, despite the known importance of timing of exposure in mouse models and vertical transmission in insects, to our knowledge there are no studies to date that test the importance of timing of non-pathogenic microbial exposure on microbiome establishment or immune function in plants, and few in invertebrates. Would a seedling exposed to beneficial microbes mount as strong of a response as an older plant? And would exposure of otherwise sterile adult plants result in the same successional dynamics of microbiome establishment as has been observed in seedlings of some plant species (93, 178, 179)? Given that we know resistance to pathogens can change throughout the life cycle of a plant, research focused on age-related tolerance and recruitment of beneficial symbionts and plant-growth promoting bacteria has large implications in agricultural practices, such as seed treatment, greenhouse germination, and age-structured planting.

Vertical transmission also ensures stable associations between hosts and their microbiomes over evolutionary time and, therefore, sets the stage for long-term coevolution and even co-speciation. There is good evidence for vertical transmission of microbiota through gametes, secretions, or birth/delivery from across systems, but how this relates to coevolution between microbiota and their hosts remains to be determined. Long-term associations between hosts and microbiota can be uncovered through examination of co-speciation events, and these have been described in insects, such as aphids (51, 52), social bees (31), and termites (40). Furthermore, recent evidence from the hominid phylogeny also strongly supports this phenomenon (180). However, in plant systems, the current evidence is limited to a few pairwise host–symbiont interactions (181, 182). To understand the ways in which microbiota–immunity interactions influence stable association, transmission, and potentially coevolution in organisms such as plants, it may be wise to start by looking for similarities in established examples, such as the reduced genomes of symbionts commonly found in insect symbionts (183), nutritional dependence on symbionts, or physical partitioning of microbiota within the host.

Another area of advancement involves taking into account the whole suite of microbiomes associated with hosts. Despite what we know about spatially distinct microbiota in humans (5) and plants (184, 185), there are still large biases toward the below-ground (rhizosphere) microbiota of plants and the gut microbiota of vertebrates and insects. As more multi-tissue microbiome studies are generated across systems, we will be in a better position to uncover general patterns of potential cross talk among microbiomes within a host, differences in the types of pathogens being protected against across tissues, and perhaps even the role of distinct microbiomes in shaping tissue tropism of pathogens. Furthermore, parallel studies of spatially distinct microbiomes in insects could offer nice insight into, for example, the roles of internally versus externally colonizing microbiota in shaping disease susceptibility, as well as how the host immune response regulates multiple microbiomes simultaneously.

Finally, the field is still limited by challenges in data interpretation for large, complex, and dynamic microbiome systems, explaining many of the open questions regarding heritability, temporal dynamics, and co-speciation (highlighted in Table 1). However, addressing these questions is increasingly feasible through rapidly advancing sequencing and bioinformatics approaches and the compilation of biologically representative synthetic communities. Although we are still some way from having large cross-system comparative microbiome studies, as sequencing costs continue to fall and data standardization across studies becomes more stringent, such meta-analyses will likely uncover larger “rules” of microbiome assembly, diversity, and interplay with host immunity. For example, plant-microbiome literature has forged the way in our understanding of how host genetics versus environment contribute to shaping the adult microbiome [e.g., Ref. (90, 186)], and recent work from humans now raises the question of whether similar rules are true for vertebrates (187). Another, more reductionist, approach for testing fundamental predictions about microbiome establishment genetic underpinning and immune system interactions is using synthetic microbiomes, as has been well-developed in plants (86, 90, 101, 132, 188). For example, a recent study in *D. melanogaster* explored colonization of gnotobiotic flies with specific strains of bacteria to document how host genotype influences microbial abundance levels (65). Though far from painting a complete picture, approaches such as this may also provide a means to study specific microbial adaptations to the immune systems of hosts across environmental conditions and genotypes. In conclusion, as we accumulate more data across systems, we can take more comparative and/or phylogenetic approaches to better understand the evolution of microbiome–immune system interaction mechanisms and to uncover conserved microbiome-mediated immune functions across systems. Such research has broad application to both human and agricultural health and is critical in light of the emergence of antibiotic and chemical-resistant pathogens and the common use of interventions that disrupt host-microbiome associations across systems.

## Author Contributions

NM and BK both contributed to the development of ideas and writing of this manuscript.

## Conflict of Interest Statement

The authors declare that the research was conducted in the absence of any commercial or financial relationships that could be construed as a potential conflict of interest.
